# The Use of Serum Scoring Systems in Predicting Liver Fibrosis Caused by Chronic Hepatitis B: A Retrospective Case-Control Study

**DOI:** 10.3390/medicina61081490

**Published:** 2025-08-20

**Authors:** Müge Özgüler, Samet Durak, Özgen Arslan Solmaz, Gülden Eser Karlıdağ, Ömür Gündağ, Yasemin Kırık, Büşra Tanır, Sümeyye Selim Kara

**Affiliations:** 1 Department of Infectious Diseases and Clinical Microbiology, Elazığ Fethi Sekin City Training, and Research Hospital, University of Health Sciences, 23280 Elazıg, Turkey; drsametdurak@gmail.com (S.D.);; 2 Department of Pathology, Elazığ Fethi Sekin City Training, and Research Hospital, University of Health Science, 23280 Elazıg, Turkey; 3 Clinic of Infectıous Diseases and Clinical Microbiology, Elazığ Fethi Sekin City Hospital, Ministry of Health, 23280 Elazıg, Turkey

**Keywords:** fibrosis prediction, KING’s score, AGAP score, GUCI score, FIB-4, APRI, S-INDEX

## Abstract

*Background and Objectives*: Early diagnosis and monitoring of liver fibrosis in chronic hepatitis B are crucial for effective disease management and prognosis. Traditionally, percutaneous liver biopsy has been regarded as the gold standard for assessing the degree of fibrosis histopathologically. However, this method has several drawbacks. Consequently, non-invasive serum scoring systems are becoming increasingly preferred. These serum scoring systems have emerged as valuable non-invasive tools for predicting liver fibrosis in patients with chronic hepatitis B. Multiple serum-based scoring systems have been developed and validated for this purpose. The aim of this study is to determine the role of serum scoring systems in chronic hepatitis B, evaluate their performance, and analyze their correlation with liver biopsy results. *Materials and Methods*: Patients diagnosed with Chronic Hepatitis B who underwent liver biopsy and were found to have liver fibrosis associated with chronic hepatitis B between August 2018 and July 2024 were included in this retrospective comparative case-control study and liver function tests, INR, alpha-fetoprotein levels, hemogram parameters, kidney function tests, and cholesterol levels at the time of biopsy were recorded. *Results*: The present study included a total of 249 patients, comprising 138 men (55.5%; mean age 42.1 years) and 111 women (44.5%; mean age 45.8 ± 13.5 years). The results of sixteen commonly used scoring systems in the current literature were evaluated for predicting fibrosis. According to ROC analysis, the most notable score identified was the KING score (0.775). The subsequent scores, in order, were AGAP (0.768), GUCI (0.748), FIB-4 (0.735), APRI (0.729), and S-INDEX (0.701). *Conclusions*: Non-invasive methods offer potential advantages over liver biopsy. While these scoring systems demonstrate good accuracy in identifying advanced fibrosis and cirrhosis, their performance in detecting mild to moderate fibrosis is generally less reliable. They can function as preliminary screening tests to identify patients who may require further evaluation or to prioritize individuals for more advanced imaging studies or liver biopsy.

## 1. Introduction

Early diagnosis and monitoring of liver fibrosis are important in chronic hepatitis B (CHB) for effective disease management and prognosis [1]. Traditionally used, percutaneous liver biopsy is regarded as the gold standard for assessing the degree of fibrosis histopathologically. The ISHAK score evaluates the extent of fibrosis on a scale from 0 to 6, as follows: Stage 0: No fibrosis; Stage 1: Fibrous expansion in some portal areas; Stage 2: Fibrous expansion in most portal areas; Stage 3: Fibrous expansion in most portal areas with occasional portal-to-portal bridging; Stage 4: Fibrous expansion into portal areas with significant bridging; Stage 5: Significant bridging with rare nodules (incomplete cirrhosis); Stage 6: Cirrhosis [2].

However, percutaneous liver biopsy has several drawbacks, including being an invasive procedure, the possibility of sampling errors, risks of complications, patient anxiety, and potential delays in treatment. Therefore, non-invasive serum scoring systems are becoming increasingly preferred [3].

Serum scoring systems have emerged as valuable non-invasive tools for predicting liver fibrosis in patients with CHB [4]. Multiple serum-based scoring systems have been developed and validated for this purpose. These non-invasive methods offer potential advantages over liver biopsy, such as a reduced risk of complications and the ability to perform repeated assessments over time. They are also more cost-effective and can be conducted more frequently, enabling serial assessments to monitor disease progression or treatment response over time [5,6,7]. These systems have demonstrated varying degrees of effectiveness in assessing the severity of liver fibrosis. While these scoring systems demonstrate good accuracy in identifying advanced fibrosis and cirrhosis, their performance in detecting mild to moderate fibrosis is generally less reliable. The effectiveness of serum scoring systems can be influenced by age, gender, and ethnicity. The cutoff values specific to the population are uncertain. Additionally, the presence of other liver diseases, such as non-alcoholic fatty liver disease or alcohol-related liver disease, can impact the accuracy of these scoring systems, potentially leading to false positives or false negatives. There are many studies related to this topic that examine the effectiveness of scoring systems and score calculations in predicting fibrosis [4,5,6,7,8,9,10,11,12,13,14,15,16,17].

As no single test can provide a definitive diagnosis, clinicians should take into account factors such as the patient’s medical history, physical examination findings, viral load, and results from imaging studies like ultrasound or transient elastography when evaluating liver fibrosis [18].

The aim of this study is to determine the role of serum scoring systems in CHB, evaluate the performance of these systems, and analyze their correlation with liver biopsy results.

## 2. Materials and Methods

Patients diagnosed with Chronic Hepatitis B who underwent liver biopsy and were found to have liver fibrosis associated with chronic hepatitis B between August 2018 and July 2024 were included in this retrospective comparative case-control study. The study protocol received approval from the local ethics committee. (Ethical approval date and number: 20 February 2025-2025/4-9).

### 2.1. Inclusion Criteria

The inclusion criteria for this retrospective case-control cohort study are defined as follows: Patients must be over 18 years old, have undergone a liver biopsy for the diagnosis of chronic hepatitis B, and have been evaluated for fibrosis by the pathology clinic. Additionally, there should be no co-infections such as HCV or HDV that could impact liver fibrosis, no acute hepatitis diagnosis at the time of the liver biopsy, and no diagnosis of acute exacerbation of chronic hepatitis at the time of the liver biopsy. Furthermore, patients must not have concurrent malignancy, must not be receiving immunosuppressive treatment, must not have HIV co-infection, must not be using medication for any chronic disease, must not have any additional conditions that could lead to chronic liver ischemia, must not have a history of direct surgical intervention on the liver, and must have the necessary tests available for serum scoring systems at the time of biopsy.

### 2.2. Exclusion Criteria

Individuals under 18 years old, those who have not undergone a liver biopsy for chronic hepatitis B diagnosis, those with co-infections such as HCV or HDV that impact liver fibrosis, those with a preliminary diagnosis or diagnosis of acute hepatitis at the time of liver biopsy, those with a preliminary diagnosis or diagnosis of acute exacerbation of chronic hepatitis at the time of liver biopsy, individuals with concurrent malignancy, those receiving immunosuppressive treatment, individuals with HIV co-infection who are receiving treatment for it, those using medication for any chronic disease, individuals with additional diseases that may cause chronic liver ischemia (such as Cardiac Heart Failure (CHF), cardiac rhythm disorders, arterial ischemia, etc.), those with a history of direct surgical intervention on the liver, and those for whom the tests required for serum scoring systems were unavailable at the time of biopsy were excluded.

### 2.3. Data Collection

Demographic information, such as age, gender, etc., of the patients, along with HBsAg, Anti-HBs, HBeAg, Anti-HBe, HBV DNA levels, liver function tests, INR, alpha-fetoprotein levels, hemogram parameters, kidney function tests, and cholesterol levels at the time of biopsy, were recorded. Hepatobiliary ultrasound findings, hepatic steatosis status, histological activity index score (HAI), and fibrosis stages from the pathology report were analyzed. According to the Ishak score, fibrosis degrees ≥ F3, ≥F4, and F ≥ 5–6 were considered significant fibrosis, advanced fibrosis, and cirrhosis, respectively.

The scores of commonly used systems in the current literature, including APRI (AST to Platelet Ratio Index), LOK Score, FORNS Score, FIB-4 Score, FI (Fibrosis Index) Score, FibroAlpha Score, KING Score, Bonacini Score, AGAP Score, GPR (Gamma-Glutamyl Transpeptidase to Platelet Ratio) Score, AAR (AST to ALT Ratio) Score, GUCI (Göteborg University Cirrhosis Index) Score, ALBI (Albumin-Bilirubin) Score, FCI (Fibrosis Cirrhosis Index) Score, Fibro-Q Score, and S-Index, were calculated according to the literature [4,5,6,7,8,9,10,11,12,13,14,15,16,17].

### 2.4. Evaluation of Liver Fibrosis

Histopathological evaluation of liver biopsies was conducted prior to the treatment of hepatitis B. Staging and grading of liver histopathology were performed according to the modified Ishak protocols. The ISHAK score assesses the degree of fibrosis on a scale from F0 to F6. (F0: No fibrosis, F1: Fibrous expansion in some portal areas, F2: Fibrous expansion in most portal areas, F3: Fibrous expansion in most portal areas with rare portal-portal bridging, F4: Fibrous expansion into portal areas with significant bridging, F5: Significant bridging with rare nodules (incomplete cirrhosis), F6: Cirrhosis) [2]. A degree of fibrosis ≥ F3 is considered significantly fibrotic, ≥F4 is regarded as advanced fibrosis, and ≥F5 is classified as cirrhotic.

### 2.5. Noninvasive Serum Scoring

The test results from blood samples collected during the liver biopsy were reviewed, and, for this study, platelet count, alanine aminotransferase (ALT), aspartate aminotransferase (AST), bilirubin, albumin, alkaline phosphatase, gamma-glutamyl transferase, cholesterol, and prothrombin international normalized ratio (INR) were recorded. APRI (AST to Platelet Ratio Index), LOK Score, FORNS Score, FIB-4 Score, FI (Fibrosis Index) Score, FibroAlpha Score, KING Score, Bonacini-cirrhosis discriminant score (CDS) Score, AGAP Score, GPR (Gamma-Glutamyl Transferase to Platelet Ratio) Score, AAR (AST to ALT Ratio) Score, GUCI (Göteborg University Cirrhosis Index) Score, ALBI (Albumin-Bilirubin) Score, FCI (Fibrosis Cirrhosis Index) Score, Fibro-Q Score, and S-Index were calculated according to the published or patented formulas [4,5,6,7,8,9,10,11,12,13,14,15,16,17].

### 2.6. Statistical Analysis

All statistical analyses were conducted using the Statistical Package for Social Sciences (SPSS) version 23.0 (IBM Corporation; Chicago, IL, USA) and R programming version 4.4.1. We utilized frequencies (percent) for categorical variables and mean ± standard deviation [or median (minimum–maximum)] for metric variables. The normality of two groups was assessed using the Shapiro–Wilk test, while the normality of more than two groups was evaluated using the Kolmogorov–Smirnov test. Differences between groups were analyzed using the independent t-test for normally distributed data. In cases of non-normal distribution, the Mann–Whitney U test was employed. The Chi-Square test was used to evaluate the statistical data of two categorical groups. Results with *p* < 0.05 were considered statistically significant, and the confidence intervals (95% CI) for significant data were presented. The comparison of score averages among the mild fibrosis (F0–F2), moderate fibrosis, and advanced fibrosis (F3–F6) categories was performed using the Mann–Whitney U test. The correlation between scores and fibrosis was analyzed using the Pearson correlation test when the data followed a normal distribution and the Spearman correlation test when the data did not follow a normal distribution.

ROC analysis was employed to establish the cut-off values for differentiating fibrotic conditions based on the parameters APRI, LOK, FORNS, FIB-4, FI, FIBROALPHA, KING’s, BONACINI-CDS, AGAP, GPR, AAR, GUCI, ALBI, FCI, FIBROQ, and S-INDEX. A significance level of *p* < 0.05 was deemed significant.

## 3. Results

A total of 249 patients (138 men, 55.5%; mean age 42.1 ± 12.4 years; 111 women, 44.5%; mean age 45.8 ± 13.5) were included in the present study. The demographic data of the patients are presented in Table 1.

The primary endpoint of this study is to evaluate whether the scores effectively indicate fibrosis, while the secondary endpoint is to examine whether the scores correlate with the progression of fibrosis. Scoring results have been assessed according to the ISHAK fibrosis stage. Fibrosis stages have been categorized as Fibrosis Stage F0–F3 and Fibrosis Stage F4–F6. It has been noted that the scores of APRI, FIB-4, FibroAlpha, KING’s, Bonacini (CDS) index, AGAP, GPR, FCI, Fibro-Q, and S-Index significantly increase as the degree of fibrosis advances. A positive correlation has been observed between fibrosis stages and scores. However, the differences between fibrosis groups in LOK, FI, AAR, and ALBI scores were not found to be significantly correlated. The findings are presented in Table 2.

Due to the low number of patients with a high level of fibrosis, regrouping was conducted based on the degree of fibrosis. The F0–F2 and F3–F6 patient groups were re-evaluated regarding the correlation of scores with fibrosis.

The correlations of scores with fibrosis among groups categorized by patient numbers are presented in Table 3.

In this study, 16 scoring systems that were evaluated for their ability to predict liver fibrosis were analyzed using ROC analysis. Among these 16 scores, it was found that 14, excluding the AAR and ALBI scores, could statistically predict fibrosis. In our study, scores with an AUROC value greater than 0.7 were considered good indicators of fibrosis. The AUROC value, cut-off value, sensitivity, specificity, positive predictive value (PPV), and negative predictive value were determined, and the findings were presented in Table 4. Accordingly, the most significant score was identified as the KING’s score (0.775). The subsequent scores, in order, were AGAP (0.768), GUCI (0.748), FIB-4 (0.735), APRI (0.729), and S-INDEX (0.701).

**Figure 1:**
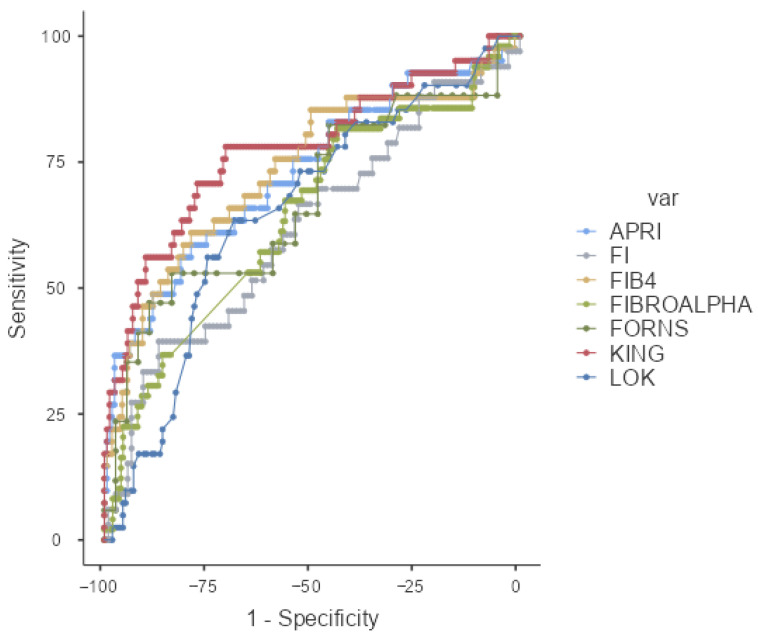
ROC curve of APRI; FI, FIB4, FIBROALPHA, FORNS, KING, and LOK scores.

**Figure 2:**
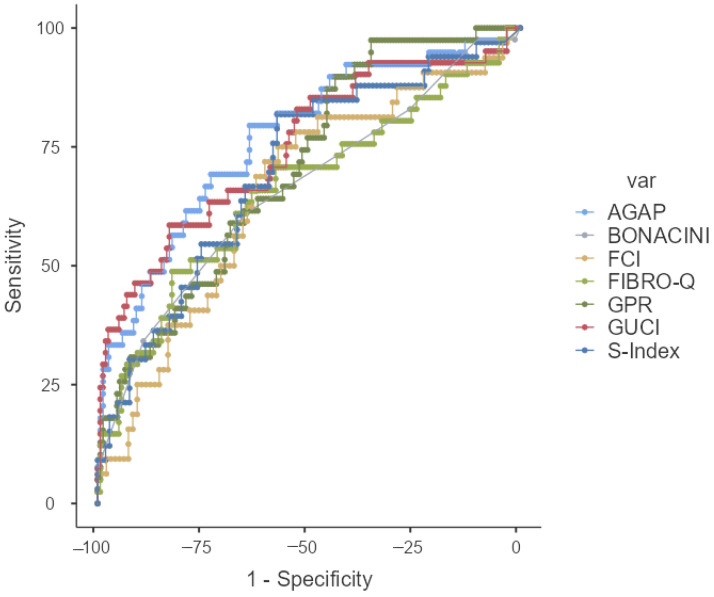
ROC curve of AGAP, BONACINI, FCI, FIBROQ, GPR, GUCI, and S-INDEX.

The ROC curves of the scoring system and AUROC have been presented in Figure 1 and Figure 2.

## 4. Discussion

In this study, 16 scoring systems that were considered to predict liver fibrosis were analyzed in treatment-naive patients with CHB. Among these 16 scores, 14 scores, except for AAR and ALBI scores, could statistically predict fibrosis. In our study, scores with an AUROC value greater than 0.7 were evaluated as good indicators of fibrosis. According to our results, the most valuable score was the KING’s score (0.775). The following scores were obtained: AGAP (0.768), GUCI (0.748), FIB-4 (0.735), APRI (0.729), and S-INDEX (0.701).

Recent studies have evaluated the effectiveness of noninvasive biomarkers in predicting liver fibrosis in patients with chronic hepatitis B (CHB). A systematic review and meta-analysis assessed the diagnostic accuracy of various noninvasive tests for detecting significant fibrosis and cirrhosis in patients with CHB. The analysis revealed that these biomarkers demonstrated moderate sensitivity and specificity, suggesting their utility as initial assessment tools for liver fibrosis in CHB [19].

Furthermore, a comprehensive review discussed the clinical application of serum and imaging-based non-invasive tests in patients with CHB. This study emphasizes that liver biopsy remains the gold standard. Non-invasive tests like APRI and FIB-4 offer valuable, non-invasive alternatives for fibrosis assessment, aiding in treatment decisions and prognostic evaluations [20].

In our study, a significant AUC value (0.729) was found for the APRI variable in distinguishing fibrotic cases (*p* < 0.001). An APRI value of ≥0.29 indicates fibrosis. The sensitivity was 56.10%, the specificity was 81.60%, the positive predictive value (PPV) was 43.40%, and the negative predictive value (NPV) was 88.08%. Recent studies have evaluated the efficacy of APRI in predicting liver fibrosis in patients with chronic hepatitis B (CHB). A previous study involving 101 CHB patients found that an APRI cut-off value of 0.49 predicted significant fibrosis with a sensitivity of 54%, specificity of 93%, and a negative predictive value of 94%, indicating good accuracy in excluding significant fibrosis [21]. Although our cutoff value for APRI was lower than that in this review, its sensitivity was found to be similar. The lower cutoff value may also explain the lower specificity. Likewise, the negative predictive value was similar to that reported in the literature. Another study demonstrated that APRI scores increased progressively with advancing fibrosis stages, achieving an area under the receiver operating characteristic curve (AUC) of 0.81 in distinguishing significant fibrosis (F2-F4) from none to minimal fibrosis (F0-F1), suggesting that APRI is a reliable noninvasive marker for assessing liver fibrosis in CHB patients [22]. Rungta et al. [23] conducted an evaluation of the APRI score’s performance, determining the AUC to be 0.756 (0.714–0.797) for ≥F2 fibrosis and 0.818 (0.776–0.861) for the F4 fibrosis group. The authors highlighted the efficacy of the APRI score in diagnosing fibrosis. In our study, it was similarly demonstrated that APRI scores progressively increased with advancing fibrosis stages, with an area under the ROC curve of 0.729 for distinguishing significant fibrosis (≥F3) from minimal or no fibrosis (<F3). This finding aligns with existing studies that suggest that APRI is a reliable, non-invasive marker for assessing liver fibrosis in patients with CHB.

In our study, we assessed the LOK scores of 198 patients diagnosed with chronic hepatitis B (CHB). The analysis revealed an AUC value of 0.654 for the LOK variable in differentiating fibrosis (*p* = 0.002). A LOK score threshold of ≥0.39 was indicative of fibrosis. The sensitivity was calculated at 63.41%, specificity at 68.79%, with a positive predictive value (PPV) of 34.67% and a negative predictive value (NPV) of 87.80%. Wang et al. [24] reported that the AUC of the LOK score was determined to be 0.796, with a cutoff value of 0.46 for predicting cirrhosis. In the present study, the AUC of the LOK score for distinguishing between F0-F1 and F2-F4 stages was presented as 0.744. In another study, the AUC of the LOK score was determined to be 0.765 (0.678–0.851) and was found to be statistically significant (*p* < 0.001). When the cut-off value was set at 0.385, the sensitivity and specificity of the LOK score were 73% and 68.6%, respectively [11]. Our results are similar to the statistical significance of the AUC of the LOK score (*p* = 0.002). A lower AUC for the LOK score was observed in our study, which may be related to factors such as the number of patients studied and the homogeneity of the fibrosis groups. The cutoff value that we determined in our study was similar to that in this study. Although the sensitivity we found for the LOK score was lower, the specificity was similar to that in this study.

We calculated FORNS scores in 54 patients with CHB. We found a significant AUC value (0.673) for the FORNS variable in distinguishing fibrosis (*p* = 0.042). A FORNS cut-off value of ≥4.91 indicates fibrosis. The FORNS score had a sensitivity, specificity, PPV, and NPV of 52.94%, 83.78%, 60%, and 79.49%, respectively. In one study, the AUROC for the FORNS score for fibrosis and cirrhosis were 0.719 and 0.872, respectively [25]. Nishikawa et al. [26] determined the FORNS score and presented their results as follows: the AUC of the FORNS index was 0.728, sensitivity was 72.0%, specificity was 65.0%, PPV was 34.0%, and NPV was 90.3%, respectively. Our results for the AUC of the FORN score were similar. In addition, according to our results, a higher PPV and lower NPV for FORN scores were observed when compared to this study. This may be related to the diversity and number of study populations.

In our study, the FIB-4 index was calculated for 204 patients with CHB. A significant AUC value (0.735) was found for FIB-4 in distinguishing fibrosis *p* < 0.001). A FIB-4 cut-off value of ≥ 1.23 indicates fibrosis. The sensitivity, specificity, PPV, and NPV were 60.98%, 79.14%, 42.37%, and 88.97%, respectively. Rugita et al. [23] found that the AUC of FIB-4 was 0.753 and 0.851 for ≥F2 and F4, respectively. To identify patients with fibrosis (≥F2), the cutoff lower value used for FIB-4 was 1.45 in this study. The sensitivity and specificity of a lower cut-off for FIB-4 (1.45 for ≥F2) were presented in this study as follows: 89% and 42%, respectively. Our results for the AUC of FIB-4 were similar to those of this study. However, we have lower sensitivity and higher specificity than those reported in this study. Our study included 204 patients with CHB for the determination of FIB-4. However, in a previous study, 520 CHB patients were identified. These different results may be explained by differences in the number of patients, patient characteristics, and differences between fibrosis-related subgroups. Previously, it was reported that the AUC, sensitivity, and specificity of FIB 4 were 0.774, 67.6%, and 73.7%, respectively [11]. Wang et al. [24] presented AUROC, sensitivity, specificity, PPV, and NPV as 0.805, 72.7%, 75.7%, 50.1%, and 89.2%, respectively. Compared with this study, our FIB-4 sensitivity value was lower than that reported in the literature, while the specificity value was higher. The PPV was lower, but the NPV was similar. This discrepancy may be due to differences in the fibrosis subgroups included in this study.

In our study, the Fibrosis Index (FI) was calculated for 140 patients with chronic hepatitis B. A significant AUC value (0.619) was found for the FI and was significant in distinguishing fibrosis (*p* = 0.040). Sensitivity, specificity, PPV, and NPV were 39.39%, 86.92%, 48.15%, and 82.30%, respectively. In a study, the cut-off values for FI < 2, AUROC, sensitivity, specificity, PPV, and NPV were 0.720, 52.7%, 83.2%, 72.1%, and 68.1%, respectively, for significant fibrosis in 228 HBV patients [27]. Our results are different from those of a study conducted in Turkey. This situation may be explained by the number of fibrosis grades included in this study. Previously, FI was evaluated in 242 patients with CHB and showed good accuracy in diagnosing significant fibrosis (≥F2), advanced liver fibrosis (≥F3), and cirrhosis (F4), with an AUC greater than 0.7. The AUC value of the fibrosis index for diagnosing significant fibrosis (≥F2) was 0.767. The AUC value of the fibrosis index for diagnosing advanced liver fibrosis (≥F3) was 0.755, and that for diagnosing cirrhosis (F4) was 0.782 (*p* < 0.001) [28]. Similar to this study, we too discovered a strong correlation between FI and fibrosis.

The FIBROALPHA score was shown to have a significant AUC value (0.643) in discriminating fibrosis (*p* = 0.002). Fibrosis was present when the FIBROALPHA score was ≥1.26. Positive predictive value (PPV) was 26.32%, negative predictive value (NPV) was 90.72%, sensitivity was 81.63%, and specificity was 44%. Nonspecific serum AFP increase occurs in from 15% to 58% of patients with chronic hepatitis and from 11% to 47% of cirrhotic cases [29]. There are no more studies on Fibroalpha score in chronic hepatitis B without HCC in the literature. A study involving 405 HBV-HCC patients found no significant AUROC level (*p* = 0.468). This study approved a cut-off value of 1.35 for Fibro ALPHA [30].

In this study, we estimated the KING score for 202 chronic hepatitis B patients. The KING variable showed a significant AUC value of 0.775 in differentiating fibrosis (*p* < 0.001). A KING cut-off score of ≥5.21 indicates fibrotic conditions. The sensitivity was 78.05%, the specificity was 70.81%, the positive predictive value was 40.51%, and the negative predictive value was 92.68%. It is emphasized that the KING’s score has been proven capable of measuring liver fibrosis [12,31]. Ekin et al. [12] investigated 1454 treatment-naive patients, and the AUROC values for KING’s Score of Significant fibrosis (Fib3–6), Advanced fibrosis (Fib4–6), and Cirrhosis (Fib5–6) were 0.725, 0.787, and 0.844, respectively. The cut-off for KING scores greater than 6.88 had a sensitivity of 60%, specificity of 75.3%, PPV of 31.72%, and NPV of 90.76% [12]. The findings of this study, conducted in Turkey, are identical to ours. In a study, the AUC of King’s score was provided as 0.765 when the cut-off value was accepted as 13.004 [32]. The sensitivity and specificity of King’s were 66% and 76%, respectively. The NPV was 93%, and the PPV was 50% [32]. In another study, the AUROC value of the KING’S Score was 0.723, with a cut-off value of 8.67. The sensitivity and specificity of the KING’s score were 50.52% and 87.26%, respectively [33]. Our analysis revealed that KING’s score demonstrated a higher AUROC than each of the other 15 scoring methods for predicting fibrosis. Our findings are consistent with previous research on the KING score.

The BONACINI-Cirrhosis discriminant score (CDS) was obtained for 198 patients. The BONACINI score has a significant AUC value (0.660) for fibrosis detection (*p* = 0.002). A BONACINI score of ≥5 was related to fibrosis. The sensitivity was 60.98%, the specificity was 64.97%, the PPV was 31.25%, and the NPV was 86.44%. In a prior study, the AUROC of the Bonacini CDS score was provided as 0.700. When the cut-off was accepted as 3, the sensitivity, specificity, PPV, and NPV were 70%, 59.5%, 27.2%, and 90.2%, respectively [4]. In another study, Bonacini CDS was assessed in 158 CHB patients, and the AUC for Bonacini CDS was provided as 0.598 [34]. Eminler et al. [35] reported that the AUC value of Bonacini-CDS was 0.646 in CHB. Kalkan et al. [36] concluded that the AUC value of Bonacini-CDS was 0.880 in CHB. We concluded that the Bonacini CDS is a poor scoring system for fibrosis differentiation in CHB.

In our research, the AGAP score was assessed in 192 patients. The AGAP score has a significant AUC value (0.768) for detecting fibrosis (*p* < 0.001). An AGAP value of ≥3.37 indicates fibrosis. The sensitivity was 79.49%, the specificity was 64.05%, the PPV was 36.05%, and the NPV was 92.45%. Ökdemir et al. [11] identified the AGAP score as having the greatest AUC value compared to other scores (AUC = 0.803). In this research, the AGAP cut-off was 4.038, with sensitivity, specificity, PPV, and NPV of 75.7%, 73.7%, 95.1%, and 29.6%, respectively. Özçelik et al. [37] found an AUROC value of 0.731 for AGAP. Accepting an AGAP cut-off of ≥1.25 results in 50% sensitivity, 93.10% specificity, 71.43% PPV, and 84.38% NPV. In a further study, the AGAP score had 47.2% sensitivity, 100% specificity, 100% PPV, 79.3% NPV, and an AUC of 0.736 [38]. In our study, the AUROC value of the AGAP score was lower than that reported in Ökdemir’s study but was nearly similar to that in Özçelik’s study.

In our study, GPR was obtained for 192 cases. The GPR score showed a significant AUC value (0.705) in differentiating fibrosis (*p* < 0.001). A GPR score of ≥0.14 implies a fibrotic state. The sensitivity was 89.74%, the specificity was 43.79%, the PPV was 28.93%, and the NPV was 94.37%. In previously published research, the results of GPR indicated the largest AUC (0.731) [39]. Ekin et al. [12] demonstrated that GPR had AUROC scores of 0.721 for significant fibrosis (F3–F6), 0.796 for advanced fibrosis (F4-F6), and 0.851 for cirrhosis (F5–F6). In this study, 1454 treatment-naive individuals underwent non-invasive examinations. Sensitivity, specificity, PPV, and NPV values were 52–75%, 85–89%, 16–40%, and 90–99%, respectively. Ding et al. [40] assessed 1.622 treatment-naïve CHB patients for GPR. However, hepatic fibrosis has been measured using the Scheuer scoring system as follows: S0 (no fibrosis), S1 (mild fibrosis without septa), S2 (moderate fibrosis with few septa), S3 (severe fibrosis with numerous septa but no cirrhosis), and S4 (cirrhosis) [41]. The AUC for GPR in S2–4, S3–4, and S4 were 0.770, 0.780, and 0.830, respectively. The sensitivity of GPR in the S2–4, S3–4, and S4 groups was 71.4%, 73.0%, and 80.0%, respectively, while the specificity was 70.8%, 72.6%, and 71.4%. The PPV and NPV of GPR in the S2–4, S3–4, and S4 groups were 76.2–65.5%, 56.2–84.9%, and 42.8–93.1%, respectively. The AUROC value of GPR in our study, found to be similar to that reported in previous studies. The PPV value for diagnosing advanced fibrosis is low, while the NPV value is significant, as expected in the literature. Furthermore, while the sensitivity was found to be high, consistent with the literature when a cutoff of 0.14 was chosen, the specificity was much lower than stated. This could be because our study had fewer cases of advanced fibrosis and cirrhosis, as well as a smaller overall patient population than earlier studies.

In our research, the GUCI score was determined for 200 cases. The GUCI score exhibited a significant AUC value (0.748) for differentiating fibrosis (*p* < 0.001). A GUCI score of ≥0.29 implies fibrosis. The sensitivity was 58.54%, the specificity was 83.02%, the positive predictive value was 47.06%, and the negative predictive value was 88.59%. In a previously reported study, 234 CHB patients were identified for noninvasive scoring methods. The AUC value for GUCI was found to be 0.744 [42]. Erdoğan et al. [43] reported GUCİ’s AUC value of 0.670. In a 2018 study conducted in China by Dong et al. [44], 30 different noninvasive fibrosis indicators were examined, and GUCI was found to be one of the three markers with the best efficiency in predicting cirrhosis in treatment-experienced CHB patients (AUC = 0.807) [44]. In treatment-naive patients, the accuracy of predicting cirrhosis was shown to be lower (AUC = 0.730). Another study underlined that GUCI was among the scoring systems with the greatest AUC levels [45].

In this study, the FCI score was determined for 128 instances with CHB. The FCI variable demonstrated a significant AUC value (0.654) in identifying fibrotic patients (*p* = 0.009). An FCI cutoff value of ≥0.07 suggests fibrosis. The sensitivity was 71.88%, the specificity was 60.42%, the positive predictive value was 37.70%, and the negative predictive value was 86.57%. In a piece of multicenter research, 770 treatment-naïve CHB patients were assessed. The AUROC values of FCI in F ≥ 3 (225), F ≥ 4 (117), and F ≥ 5 (29) were 0.711, 0.756, and 0.793, respectively [44]. Okdemir et al. [11] assessed the FCI score in 273 patients with chronic hepatitis B. The cutoff value for FCI has been presented as 0.171. The AUROC level for FCI has been established to be 0.751. The sensitivity, specificity, positive predictive value, and negative predictive value were 70.3%, 72.9%, 93.5%, and 28.7%, respectively. We noticed that the AUC level for FCI was lower than previously reported. This is due to the distribution of fibrosis stages that were included in this study. Our sensitivity results are similar to Okdemir’s.

In our research, 200 CHB cases were assessed for FIBRO-Q. In our study, the FIBRO-Q score showed a significant AUC for fibrosis discrimination (AUC = 0.659; *p* = 0.002). A FIBRO-Q cut-off score of ≥3.32 suggests fibrotic disease. The sensitivity was 48.78%, the specificity was 82.39%, the positive predictive value was 41.67%, and the negative predictive value was 86.18%. In a prior piece of research, the FIBRO-Q score was obtained in 273 chronic hepatitis B patients. The cut-off value, AUROC level, sensitivity, specificity, PPV, and NPV were 3.226, 0.718, 67.6%, 72%, 93.4%, and 27.5%, respectively [11]. Ekin et al. [12] analyzed the FIBRO-Q score in 1454 treatment-naive CHB patients. The AUC level of the FİBRO-Q score for significant fibrosis (Fib3–6), advanced fibrosis (Fib4–6), and cirrhosis (Fib5–6) was 0.594, 0.717, and 0.843. The cut-off value, sensitivity, specificity, PPV, and NPV have been shown to be 1.07–1.73, 57.1–80%, 50.4–78.7%, 9.8–19.57%, and 87.6–99.2%, respectively, related to increased fibrosis severity. Kaya et al. [33] reported the results of a non-invasive scoring system in 1051 CHB patients. AUC values for FİBROQ score, cut off, sensitivity, and specificity were 0.595, 2.72, 24.22%, and 93.10%, respectively. We found comparable AUC values for the FIBRO-Q score to Kaya, Okdemir, and Ekin’s research.

In the course of our research, the S-INDEX was determined for 139 patients with CHB. The S-INDEX score demonstrated a significant AUC value (0.701) in identifying fibrosis (*p* = 0.001). An S-INDEX value of ≥0.04 predicts fibrosis. The sensitivity was 81.82%, the specificity was 57.55%, the positive predictive value was 37.50%, and the negative predictive value was 91.04%. In a previous study, the AUC level of the S-INDEX score for significant fibrosis (Fib3–6), advanced fibrosis (Fib4–6), and cirrhosis (Fib5–6) was 0.715, 0.783, and 0.859, respectively. The cut-off value, sensitivity, specificity, PPV, and NPV related to increased fibrosis severity were 6.28–8.65, 63.3–77.8%, 71.7–84.9%, 12.43–29.8%, and 91.4–99.2%, respectively [12]. Kang et al. [46] found that the AUC value for the S-index was 0.722 after evaluating 275 cases. The sensitivity, specificity, PPV, and NPV were, respectively, 78.7%, 57.0%, 69.9%, and 69.7%. In another study, it was stated that the S-index had the greatest AUC for predicting fibrosis among the analyzed indices in 200 CHB patients, with an AUC value of 0.810. The optimum S-index threshold for considerable fibrosis was ≥0.3 with 94% specificity and 87% PPV, while, for ruling out major fibrosis, it was <0.1 with 96% sensitivity and 91% negative predictive value. In another study, it was emphasized that the S-index was more accurate in predicting cirrhosis (91%) than in predicting advanced (79%) or substantial fibrosis (68%) [47]. S-INDEX was one of the significant indices in our analysis. Our S-INDEX findings were found to be lower than those in the literature. This could be due to the fact that the patients included in this study had normal platelet and albumin levels, as well as a lower count of cirrhotic patients than those analyzed in the current study.

Previous studies have noted the absence of affordable, effective, and easily applicable methods for detecting hepatitis B-related liver fibrosis, particularly in resource-limited settings. It has been suggested that the Forns index and S-index could serve as useful tools for identifying advanced fibrosis in HBV patients treated in community hospital settings [48].

Noninvasive serum biomarkers of hepatic fibrosis are the current research focus. There are newly identified markers in the literature that serve as indicators of fibrosis. Pentraxin-3, involved in inflammation regulation and tissue repair, was significantly lower in CHB patients than in controls, with further declines observed as fibrosis advanced. Notably, even stage 1 fibrosis showed reduced PTX-3 levels, suggesting its potential as a non-invasive marker for early fibrosis detection [49]. Mac-2 binding protein glycosylation isomer (M2BPGi) demonstrated the highest diagnostic performance. In CHB patients without notable necroinflammation, M2BPGi showed superior accuracy in detecting significant fibrosis, supporting its utility in identifying cases that may require antiviral therapy [50].

In patients with HBV-associated compensated chronic liver disease, serum Chitinase 3-like 1 (CHI3L1) levels correlated positively with histological severity, though larger studies are required to confirm this association. Recognizing the limitations of liver biopsy and the suboptimal accuracy of current noninvasive fibrosis markers in CHB, a novel diagnostic model integrating CHI3L1 and routine clinical parameters has been studied in a study. In this study, a novel model was developed by combining CHI3L1, AFP, and platelet count, which demonstrated superior diagnostic performance compared to APRI, FIB-4, and CHI3L1 alone [51].

In comparison with transient elastography (FibroScan), serum biomarkers, including hyaluronan (HA), procollagen type III N-terminal peptide (PIIINP), type IV collagen (IVC), laminin (LN), alanine aminotransferase (ALT), and aspartate aminotransferase (AST), demonstrate potential for detecting clinically significant liver fibrosis. The combination of the five serum markers is a reliable noninvasive method to predict significant liver fibrosis in patients with CHB [52].

The investigation into circulating angiotensin-converting enzyme (ACE) levels in patients newly diagnosed with chronic hepatitis B (CHB) seeks to elucidate the association between liver fibrosis and serum ACE concentrations. It has been observed that CHB patients often exhibit elevated circulating ACE levels, particularly those with more advanced stages of liver fibrosis. Integrating ACE level assessments into the clinical evaluation of CHB patients, in conjunction with other diagnostic parameters, could offer significant prognostic insights [53]. As fibrosis progresses, there is a notable decline in serum levels of platelet-derived growth factor (PDGF)-BB, indicating its potential utility as a non-invasive biomarker for determining the stage of fibrosis in individuals with chronic hepatitis B (CHB) [54].

The highest levels of butyrylcholinesterase (BChE) mRNA are found in the liver, followed by the lung and the brain [55]. BChE levels are low in systemic conditions like liver disease, renal disease, malnutrition, malignancies, and burns [56]. BChE measurement is sometimes included in the panel of liver function tests due to its hepatic origin. It is an indicator of acute hepatitis or cirrhosis of the liver [57].

These studies show that the search for biomarkers that could be effective in early detection of liver fibrosis caused by chronic hepatitis B continues, that the topic remains current, and that the pursuit of noninvasive markers or indicators and scoring systems that indicate fibrosis without risk will continue.

Our study’s limitations include the following: First, the proportion of cirrhotic patients (≥F3) is only 19.7%, while the proportion of patients with fibrosis (F0–F2) is 80.3%. This circumstance leads to group heterogeneity. Second, the upper limit of normal for ALT in this investigation was set at cutoff ALT values < 40 IU/mL. However, ALT is predicted to be 35 IU/mL in healthy men and 25 IU/mL in healthy women based on current guidelines [58]. This could lead to disparities in how the research in the literature is evaluated.

Lastly, this study’s retrospective design may introduce bias. Although our analysis included well-documented cases from databases and medical records, the vast number of patients from multicenter and prospective studies necessitates validation of our findings.

## 5. Conclusions

Serum scoring systems have emerged as valuable non-invasive tools for predicting liver fibrosis in patients with chronic hepatitis B (CHB). These non-invasive methods offer potential advantages over liver biopsy, such as a reduced risk of complications and the ability to perform repeated assessments over time. While these scoring systems demonstrate good accuracy in identifying advanced fibrosis and cirrhosis, their performance in detecting mild to moderate fibrosis is generally less reliable. Furthermore, serum scoring systems can be seamlessly integrated into routine clinical practice, providing clinicians with readily available tools to evaluate liver fibrosis risk. They can serve as initial screening tests to identify patients who may need further evaluation or to prioritize individuals for more advanced imaging studies or liver biopsy. We believe that multicenter prospective studies with similar patient numbers based on fibrosis and cirrhosis stages would be a suitable way to validate our findings.

## Figures and Tables

**Table 1:** Demographic features and laboratory findings of patients.

Patients	Mean or n (%)
Age	43 ± 13 (21–77)
Female	45.8 ± 13.5
Male	42.1 ± 12.4
**Sex**	
Female	111 (44.5)
Male	138 (55.5)
**Fibrosis**	249
ISHAK ≤ 2	200 (80.3)
ISHAK 3–4	44 (17.7)
ISHAK 5–6	5 (2)
**Histological activity İndex (HAI)**	249
HAI: 1–3	39 (15.6)
HAI: 4–6	141 (56.7)
HAI: 7–10	60 (24)
HAI: 11–15	9 (3.6)
**Platelet count (10^9^/L)**	239.61 ± 59.34
Female	262.22 ± 64.35
Male	218.33 ± 45.07
**AST (U/L)**	26.64 ± 13.39
Female	23.23 ± 6.16
Male	29.79 ± 16.89
**ALT (U/L)**	30.17 ± 38.75
Female	20.02 ± 9.02
Male	39.55 ± 51.39
**Total bilurubin (mg/dL)**	0.63 ± 0.33
Female	0.53 ± 0.21
Male	0.73 ± 0.40
**Albumin (g/L)**	41.76 ± 2.7
Female	41.42 ± 2.60
Male	42.07 ± 2.91
**INR**	1.04 ±0.08
Female	1.04 ± 0.09
Male	1.05 ± 0.08
**ALP (U/L)**	77.28 ± 24.90
Female	72.97 ± 22.32
Male	81.56 ± 26.73
**GGT (U/L)**	23.60 ± 22.69
Female	18.27 ± 13.54
Male	28.76 ± 28.04
**Cholesterol (mg/dL) (in 53 Patients)**	176.34 ± 34.70
Female (25)	186.04 ± 35.67
Male (28)	167.68 ± 31.99
**AFP μ/L**	3.85 ± 7.96
Female	4.21 ± 11.1
Male	3.52 ± 2.72
**HBV DNA IU/mL**	64,142.741 ± 301,427.605
Female	40,721.124 ± 174,142.665
Male	83,288.983 ± 374,377.896
**HBeAg negative**	230 (92.4)
Female	110 (48)
Male	120 (52)
**HBeAg positive**	19 (7.6)
Female	10 (54)
Male	9 (46)

**Table 2:** Scoring method: the number of patients can be calculated, along with mean ± SD and median score values.

Scoring Method (Number of Calculated Case)	Mean ± SD	Median (Min–Max)/%
APRI (204)	0.265 ± 0.15	0.225 (0.092–1.111)
LOK (198)	0.351 ± 0.192	0.33 (0.05–1.1)
FORNS (54)	4.028 ± 1.726	4.09 (1–8.98)
FIB-4 (204)	1.053 ± 0.564	0.912 (0.287–3.5)
FI (140)	−36.074 ± 2.768	−36.32 (−42.97–−25.72)
FIBROALPHA (249)	1.287 ± 0.143	1.302 (0.872–2.125)
KING (202)	5.408 ± 3.865	4.525 (0–26.067)
BONACINI (198)	4.232 ± 1.216	4 (1–7)
AGAP (192)	0.805 ± 1.762	0.343 (0.043–15.942)
GPR (192)	0.2 ± 0.2	0.2 (0.1–1.8)
AAR (205)	1.127 ± 0.424	1.09 (0.21–3.3)
GUCI (200)	0.274 ± 0.164	0.242 (0–1.278)
ALBI (139)	−2.881 ± 0.325	−2.913 (−3.729–−0.95)
FCI (128)	0.094 ± 0.099	0.069 (0–0.855)
FIBRO-Q (200)	2.393 ± 1.525	2.024 (0–8.254)
SINDEX (139)	0.066 ± 0.084	0.045 (0–0.603)
FIBROSIS		
ISHAK 0–2	200	80.3
ISHAK 3–4	44	17.7
ISHAK 5–6	5	2
FIBROSIS		
ISHAK < 3	200	80.3
ISHAK ≥ 3	49	19.7

**Table 3:** The correlations of scores with fibrosis among groups categorized by patient numbers.

	ISHAK < 3	ISHAK ≥ 3	Total	Test Statistics	*p* Value
*APRI*	0.22 (0.092–0.662)	0.299 (0.101–1.111)	0.225 (0.092–1.111)	1810.000	**<0.001** ^x^
*LOK*	0.29 (0.05–1.1)	0.45 (0.1–0.89)	0.33 (0.05–1.1)	2228.000	**0.002** ^x^
*FORNS*	3.68 ± 1.497	4.787 ± 1.983	4.028 ± 1.726	−2.274	**0.027** ^y^
*FIB4*	0.833 (0.287–2.577)	1.357 (0.337–3.5)	0.912 (0.287–3.5)	1773.000	**<0.001** ^x^
*FI*	−36.306 ± 2.651	−35.321 ± 3.041	−36.074 ± 2.768	−1.801	0.074 ^y^
*FIBROALPHA*	1.29 (0.872–1.846)	1.35 (1.02–2.125)	1.302 (0.872–2.125)	3498.000	**0.002** ^x^
*KING*	4.137 (0–15.639)	7.159 (2.112–26.067)	4.525 (0–26.067)	1488.000	**<0.001** ^x^
*BONACINI*	4 (1–6)	5 (1–7)	4 (1–7)	2186.000	**0.001** ^x^
*AGAP*	0.307 (0.043–8.377)	0.594 (0.072–15.942)	0.343 (0.043–15.942)	1384.000	**<0.001** ^x^
*GPR*	0.2 (0.1–1.5)	0.2 (0.1–1.8)	0.2 (0.1–1.8)	1761.000	**<0.001** ^x^
*AAR*	1.07 (0.21–3.3)	1.135 (0.39–1.83)	1.09 (0.21–3.3)	3380.500	0.903 ^x^
*GUCI*	0.217 (0–0.695)	0.306 (0.117–1.278)	0.242 (0–1.278)	1642.000	**<0.001** ^x^
*ALBI*	−2.922 (−3.729–−0.95)	−2.824 (−3.385–−2.213)	−2.913 (−3.729–−0.95)	1377.000	0.066 ^x^
*FCI*	0.062 (0–0.366)	0.092 (0–0.855)	0.069 (0–0.855)	1062.500	**0.009** ^x^
*FIBROQ*	1.883 (0–7.512)	3.056 (0.415–8.254)	2.024 (0–8.254)	2225.000	**0.002** ^x^
*SINDEX*	0.04 (0–0.302)	0.056 (0–0.603)	0.045 (0–0.603)	1046.000	**<0.001** ^x^

^x^ Mann–Whitney U test; ^y^ Independent Samples *t*-test; Median (Minimum–Maximum); Mean ± Standard Deviation.

**Table 4:** ROC analysis results for each scoring system in predicting fibrosis.

	AUC (%95 CI)	*p*	Cut-Off	Sensitivity%	Specificity%	PPV%	NPV%
APRI	0.729 (0.634–0.824)	<0.001	≥0.29	56.10	81.60	43.40	88.08
LOK	0.654 (0.560–0.747)	0.002	≥0.39	63.41	68.79	34.67	87.80
FORNS	0.673 (0.506–0.841)	0.042	≥4.91	52.94	83.78	60.00	79.49
FIB4	0.735 (0.639–0.831)	<0.001	≥1.23	60.98	79.14	42.37	88.97
FI	0.619 (0.503–0.734)	0.040	≥−33.58	39.39	86.92	48.15	82.30
FIBROALPHA	0.643 (0.553–0.734)	0.002	≥1.26	81.63	44.00	26.32	90.72
KING	0.775 (0.684–0.865)	<0.001	≥5.21	78.05	70.81	40.51	92.68
BONACINI	0.660 (0.561–0.760)	0.002	≥5.0	60.98	64.97	31.25	86.44
AGAP	0.768 (0.681–0.885)	<0.001	≥3.37	79.49	64.05	36.05	92.45
GPR	0.705 (0.619–0.791)	<0.001	≥0.14	89.74	43.79	28.93	94.37
AAR	0.506 (0.409–0.603)	0.901	-	-	-	-	-
GUCI	0.748 (0.656–0.840)	<0.001	≥0.29	58.54	83.02	47.06	88.59
ABLI	0.606 (0.494–0.719)	0.066	-	-	-	-	-
FCI	0.654 (0.543–0.765)	0.009	≥0.07	71.88	60.42	37.70	86.57
FIBROQ	0.659 (0.556–0.761)	0.002	≥3.32	48.78	82.39	41.67	86.18
SINDEX	0.701 (0.598–0.804)	0.001	≥0.04	81.82	57.55	37.50	91.04

PPV: Positive Predictive Value, NPV: Negatif Predictive Value.

## Data Availability

Data is contained within the article.
